# Maternal anemia and birth weight: A prospective cohort study

**DOI:** 10.1371/journal.pone.0212817

**Published:** 2019-03-18

**Authors:** Ana Claudia Morais Godoy Figueiredo, Isaac Suzart Gomes-Filho, Josicélia Estrela Tuy Batista, Géssica Santana Orrico, Edla Carvalho Lima Porto, Rodolfo Macedo Cruz Pimenta, Sarah dos Santos Conceição, Sheila Monteiro Brito, Michelle de Santana Xavier Ramos, Maria Cristina Ferreira Sena, Saulo Wesley Silva Lessa Vilasboas, Simone Seixas da Cruz, Mauricio Gomes Pereira

**Affiliations:** 1 Faculty of Health Sciences, University of Brasilia, Brasília, Distrito Federal, Brazil; 2 Department of Health, Feira de Santana State University, Feira de Santana, Bahia, Brazil; 3 Department of Epidemiology, Federal University of Recôncavo da Bahia, Santo Antônio de Jesus, Bahia, Brazil; Centre Hospitalier Departementai Vendee, FRANCE

## Abstract

**Objective:**

To investigate the association between maternal anemia and low/insufficient birth weight.

**Design:**

A prospective cohort study of pregnant women who underwent prenatal care at the healthcare units in a municipality of northeast Brazil together with their newborn infants was carried out. The pregnant women were classified as having anemia when the hemoglobin level was below 11 g/dl. Infants who were born full term weighing less than 2500 grams were classified as low birth weight, and those weighing between 2500 and 2999 grams were classified as insufficient weight. The occurrence of maternal anemia and its association with birth weight was verified using crude and adjusted Relative Risk (RR) estimates with their corresponding 95% confidence intervals (95%CIs).

**Results:**

The final sample was comprised of 622 women. Maternal anemia was considered a risk factor for low/insufficient birth weight, after adjusting the effect measurement for maternal age, family income, urinary infection, parity, alcoholic beverage consumption during pregnancy and gestational body mass index: RR_adjusted_ = 1.38 [95% CI: 1.07 to 1.77].

**Conclusions:**

Maternal anemia was associated with low/insufficient birth weight, representing a risk factor for the gestational outcomes studied.

## Introduction

Low birth weight has been widely studied and is an important risk factor for infant morbidity and mortality [1–4]. However, insufficient weight has received little attention [5–8], even though three decades ago, children with birth weights less than 3000 grams were considered to have a risk of mortality that was three times higher during the first year of life than that of children whose weights were above or equal to this cutoff point [9,10]. In Brazil, 2017, 8.49% of the newborn infants had weight <2500g. In this same year, the frequency of children with insufficient weight, that is ≥ 2500g and <3000g, was almost three times more 22.41%, increasing the necessity to investigate this weight special group [7].

The classic risk factors for low birth weight are associated with unfavorable biological, social and environmental conditions that may occur before or during the pregnancy period [4, 11–13]. Nutritional determinants, such as pre-gestational weight and weight gain during pregnancy, influence birth weight. Thus, inadequate maternal caloric intake, which may be the result of a diet that is nutritionally poor, leads to lower absorption of essential micronutrients, such as vitamin B12 and iron, for fetal growth [14].

Although the determinants of both low and insufficient weight at birth are similar, the mechanism that links maternal anemia to insufficient birth weight is not fully known. Few prospective cohort studies have analyzed the association between maternal anemia and low birth weight [15–25]. Indeed, after a rigorous search of previous investigations on the topic, only two retrospective cohort studies, from Colombia and Finland, that addressed the relationship between nutritional exposure and insufficient birth weight were identified [5, 6].

Given the relevance of the theme, the high frequency of insufficient birth weight in the studied group, and the knowledge that the association between maternal anemia and birth weight is affected by demographic and socioeconomic factors, an investigation of this relationship in diverse populations is necessary to identify the groups at greatest risk. The objective of this study was to verify the frequency of maternal anemia and its association with low/insufficient birth weight in users of the public health service from a population in northeastern Brazil.

## Materials and methods

### Study design/population

A prospective analytical cohort study was carried out with pregnant women, who underwent prenatal follow-up at healthcare units in the urban area of Santo Antônio de Jesus, Bahia, Brazil, and their newborns. The data collection period was from January 2013 to March 2017.

### Sample size

To calculate the sample size, the following parameters were used: an Odds Ratio of 2.36 [26], the frequency of the rarest outcome, a low-birth-weight incidence of 8.29% in the group of pregnant women without anemia [26] and a ratio of exposed *versus* not exposed to anemia of 1:3. A study power of 80%, alpha error of 5% and the 95% confidence interval were also considered. Thus, the minimum estimated sample size was 141 pregnant women in the group diagnosed with anemia and 421 in the non-anemic group. In addition, 10% was added to the sample size to correct for possible losses, with a minimum sample calculation of 618 pregnant women. The sample size was calculated using Epi Info (StatCalc), version 7 [27].

The present study was approved by the Research Ethics Committee of Feira de Santana State University. All pregnant women voluntarily participated in the study and signed the Free Consent Form.

### Eligibility criteria

#### Inclusion criteria

The pregnant women in this study had the following: pregnancy with fetal gestational ages between 8 and 32 weeks, assistance from the public health system, prenatal care from the selected healthcare units, live births, and children available for the study.

#### Exclusion criteria

Women were excluded from the study if they had a twin pregnancy, preterm birth or a history of bleeding that required hospital treatment for at least 24 hours.

### Data collection procedures

Information concerning the pregnant women was first obtained through interviews. Then, trained researchers performed blood collections, and oral examinations of all teeth were performed by a dentist according criteria defined previously [28, 29].

The data that could not be obtained during the interview were acquired from the patient's chart and/or pregnancy card. During the postpartum period, information on birth weight was collected from the birth registration document (Declaration of Live Births).

### Data collection tools

The questionnaire form was divided into six sections: 1) identification, socioeconomic-demographic data and environmental data; 2) nutritional information; 3) gynecological-obstetric history; 4) drug information; 5) variables related to the anthropometry of the pregnant woman; and 6) information concerning childbirth.

### Blood collection for laboratory tests

The blood collection followed the standard criteria of collection and storage [30] to obtain the complete blood count and ferritin dosage.

### Birth weight

Birth weight was measured immediately after delivery on a precise scale and subsequently recorded in the Declaration of Live Birth by a health professional who participated in the birth [31].

### Criteria for the definitions of exposure and outcome

#### Exposure: Maternal anemia

Study participants were diagnosed as having maternal anemia when the hemoglobin level was below 11 g/dl or when the hematocrit was less than 33% (32). Additionally, the participants were diagnosed with iron deficiency anemia when the reference value for serum ferritin was less than 15 femtoliters and the mean corpuscular volume (MCV) was less than 80 femtoliters [32]. Participants were diagnosed with anemia of chronic disease when the MCV was normal, from 80 to 96 femtoliters, and when they had the aforementioned hemoglobin level [32]. In addition, hemoglobin levels were evaluated in their continuous form.

#### Outcome: Low/Insufficient birth weight

The classification of birth weight was defined according to the criteria of the World Health Organization [2]. Children born with weights above or equal to 3000 grams were allocated to the group of newborns with satisfactory weights. Newborns with birth weights less than 2500 grams were classified as low birth weight, and those weighing between 2500 and 2999 grams were classified as insufficient weight. In addition, the birth weight was evaluated in its continuous form.

### Procedure for analyzing the data

Descriptive analysis for all selected variables was performed, according to the relative and absolute frequency. The Kolmogorov-Smirnov test [33] was applied, and histogram inspection was performed to verify the normality of the continuous variables. Student's t-test [34] or the Mann-Whitney U test [35] was used, according to the normality test of the variable, employing the mean, median and standard deviation to verify the differences between the groups. Categorical covariables were also evaluated for distribution, the presence of maternal anemia and weight less than 3000 grams using the chi-square test [36] or Fisher's exact test [37], with a significance level of 5%.

The investigation of the association between maternal anemia and low/insufficient birth weight was performed using logistic regression analysis by estimating the crude and adjusted odds ratio (OR) with their 95% confidence intervals and a significance level of 5%. Poisson model was used to convert the association measurement in relative risk (RR) with it 95% confidence interval.

Initially, a conceptual framework was adopted to select the following covariables involved in the multicausality of the association between maternal anemia and low/insufficient birth weight: maternal age, family income, urinary infection, parity, alcoholic beverage consumption during pregnancy and gestational BMI.

Subsequently, potential confounding factors and effect modification were selected by stratified analysis. The interaction identification was performed using the maximum likelihood ratio test, according to the definition of the saturated and reduced models for each of the possible effect modifier variables. A covariable was considered confounding when, after being eliminated from the saturated model, the covariable promoted a variation in the effect measurement that was greater than 10%compared to the main association measurement. In addition, the criterion of epidemiological importance for the selection of confounding covariables was adopted during modeling. The diagnosis of the model was performed using the Hosmer-Lemeshow test [38].

To evaluate the relationship between hemoglobin levels and the outcome, the multicollinearity test was performed, followed by univariate and multiple linear regression analysis using the common minimum squares technique [39], after confirming the linearity between the main variables by means of the F test [40]. For the adjusted model, the covariables that were considered to be confounders were used. These covariables included those that altered the parameters of the null model based on the statistical criteria (p <0.20) and those of epidemiological relevance. Stata software, version 15, was used for data processing and analysis [41].

## Results

**Fig 1:**
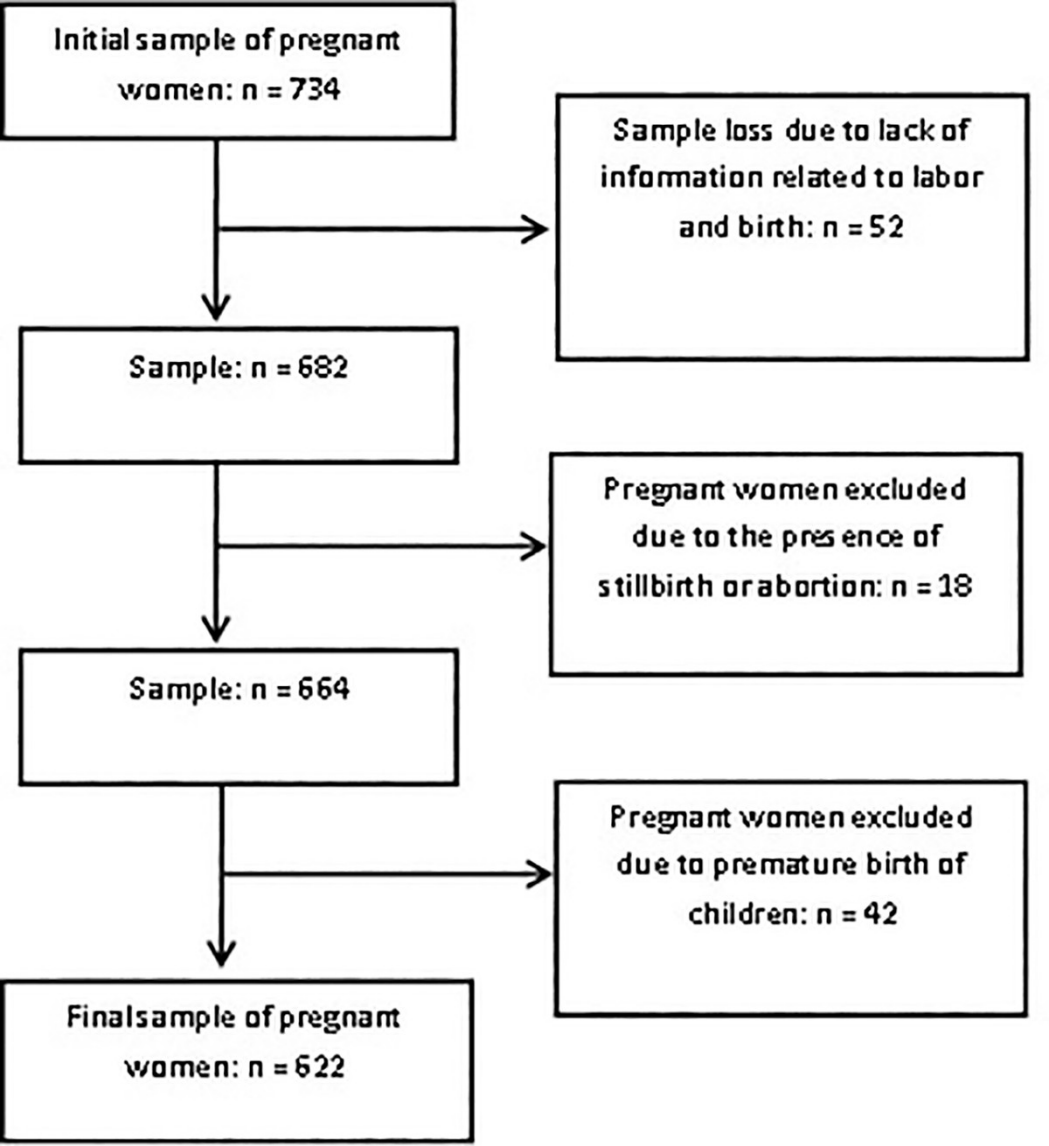
Flowchart of the sample participant selection process.

The sample consisted of 622 pregnant (Fig 1) women who utilized the public health service of the municipality of Santo Antônio de Jesus, BA. The mean age of the participants was 25.5 years (± 6.5 SD), with a median of 25 years and a range of 13 to 46 years. The refusal rate was 2%.

The pregnant women were classified into two groups per the maternal anemia diagnostic criteria: 24.9% (n = 155) with anemia and 75.1% (n = 467) without anemia. Regarding the severity of maternal anemia, 20.1% of the pregnant women were diagnosed with mild anemia, and 4.8% were diagnosed with moderate anemia; there was no record of severe maternal anemia. The frequency of iron deficiency anemia was 6.0% among the participants, whereas the frequency of anemia of chronic disease was 18.9%. Iron deficiency was present in 16.4% of the pregnant women.

The socioeconomic-demographic data are described in Table 1, and the data related to the maternal condition are shown in Table 2. Among the characteristics, only urinary infection, parity and the late onset of prenatal care showed statistically significant differences between the women with anemia and those without anemia, indicating that for most covariables, the comparison groups were homogeneous.

**Table 1:** Number (n) and percentage (%) of the socioeconomic-demographic characteristics of the sample, according to the presence of anemia. Santo Antônio de Jesus, Bahia, Brazil, 2017 (n = 622).

Maternal Anemia			
CHARACTERISTICS	Yes	No	p^†^
	n (%)	n (%)	
	155 (24.9)	467 (75.1)	
AGE (years)			
18–35	120 (24.0)	380 (76.0)	
<18	21 (33.9)	41 (66.1)	0.09
>35	14 (23.3)	46 (76.7)	0.91
EDUCATION LEVEL (years)			
≥8	108 (24.2)	338 (75.8)	
< 8	47 (26.7)	129 (73.3)	0.52
CONJUGAL STATUS			
With partner	140 (25.0)	419 (75.0)	
Without partner	15 (23.8)	48 (76.2)	0.83
RACE/SKIN COLOR			
Not black	91 (24.0)	288 (76.0)	
Black	64 (26.3)	179 (73.7)	0.51
CURRENT OCCUPATION			
Paid	65 (23.0)	218 (77.0)	
Unpaid	90 (26.6)	249 (73.4)	0.30
FAMILY INCOME*			
> 2 minimum wages	51 (27.4)	135 (72.6)	
≤ 2 minimum wages	104 (23.9)	332 (76.1)	0.35
HOUSEHOLD DENSITY (number of people per household)			
≤ 4	132 (25.8)	379 (74.2)	
>4	23 (20.7)	88 (79.3)	0.26

*Minimum wage values (per month) at the time of collection: 2013, R $ 678.00; 2014, R $ 724.00; 2015, R $ 788.00; 2016, R $ 880.00; 2017, R $ 937.00.

^†^p value: level of significance ≤ 0.05.

**Table 2:** Number (n) and percentage (%) of the characteristics related to the health and lifestyle of the sample, according to the presence of maternal anemia. Santo Antônio de Jesus, Bahia, Brazil, 2017 (n = 622).

Maternal Anemia			
CHARACTERISTICS	Yes	No	p^†^
	n (%)	n (%)	
	155 (24.9)	467 (75.1)	
SEX OF THE NEWBORN			
Male	70 (22.9)	236 (77.1)	0.25
Female	85 (26.9)	231 (73.1)	
URINARY INFECTION			
No	141 (24.1)	445 (75.9)	
Yes	14 (38.9)	22 (61.1)	0.05
PERIODONTITIS**			
No	111 (23.5)	361 (76.5)	0.68
Yes	23 (25.6)	67 (74.4)	
MATERNAL ARTERIAL HYPERTENSION			
No	151 (25.0)	454 (75.0)	
Yes	4 (23.5)	13 (76.5)	0.89
ABORTION			
No	128 (25.2)	380 (74.8)	
Yes	27 (23.7)	87 (76.3)	0.74
GESTATIONAL BMI*			
Proper weight	72 (25.3)	213 (74.7)	
Low weight	32 (27.6)	84 (72.4)	0.63
Overweight	40 (26.9)	109 (73.1)	0.72
Obese	11 (15.3)	61 (84.7)	0.08
PARITY			
> 2 children	118 (33.5)	234 (66.5)	0.01
≤ 2 children	65 (24.1)	205 (75.9)	
BEGINNING OF THE PRENATAL ACCOMPANIMENT			
≤ 3 months	128 (23.4)	419 (76.6)	0.02
> 3 months	27 (36.0)	48 (64.0)	
MATERNAL SMOKING HABIT^‡^			
No	140 (24.6)	429 (75.4)	0.89
Yes	38 (25.5)	38 (74.5)	
ALCOHOLIC BEVERAGE CONSUMPTION DURING PREGNANCY			
No	124 (25.1)	371 (74.9)	0.54
Yes	24 (22.2)	84 (77.8)	
FERROUS SALT SUPPLEMENTATION DURING PREGNANCY			
No	30 (20.0)	120 (80.0)	
Yes	125 (26.5)	347 (73.5)	0.11

* The Atalah curve was employed to calculate this covariable

** Gomes-Filho criteria for the definition of periodontitis^28^

^†^ p value: level of significance ≤ 0.05

^‡^ There were deficits in this information.

Regarding birth weight, 29.4% (183) of the participants had children with birth weights less than 3000 g, with 3.4% of the live births being classified as low birth weight and 26% being classified as insufficient birth weight. The frequency of mothers who had children of satisfactory weight was 70.6% (439). The centile for birthweight were 2940 grams, 3272 grams and 3565 grams for 25%, 50% and 75%, respectively

The central tendency measures of the descriptors used for the diagnosis of maternal anemia, according to birth weight, are summarized in Table 3. Notably, statistically significant differences were evident only for the mean values of hemoglobin (p = 0.03) and hematocrit (p = 0.02).

**Table 3:** Central tendency and dispersion measurements of the descriptors used to evaluate maternal anemia, according to the newborn weight, in users of the public health system in Santo Antônio de Jesus, Bahia, Brazil, 2017 (n = 622).

	Weight < 3000 g			Weight ≥ 3000 g			
Descriptors	Mean	±SD*	Median	Mean	±SD*	Median	p^†^
Hemoglobin level (g/dl) ^‡^	11.6	±1.1	11.6	11.8	±1.1	11.9	0.03
Red blood cell count (millions) ^‡^	4.1	±0.4	4.1	4.6	±8.7	4.2	0.14
Hematocrit (%)^‡^	35.1	±3.2	35.2	36.0	±4.2	36.3	0.02
Ferritin level (femtoliters) ^‡^	44.8	±38.9	31.8	45.2	±40.2	32.2	0.89
Mean Corpuscular Volume—MCV (femtoliters) ^‡^	85.9	±7.1	85.0	87.0	±5.5	86.4	0.06

* SD: Standard deviation

^†^ p value: level of significance ≤ 0.05

^‡^ Reference value: Hemoglobin: ≥ 11 g / dl; Blood cell count: > 4 million; Hematocrit: ≥ 33%; Ferritin: ≥ 15 femtoliters; MCV = 80–96 femtoliters.

Women diagnosed with maternal anemia showed a significantly higher incidence of children with birth weights<3000 g than the women who were not exposed to anemia during pregnancy (RR_**crude**_ = 1.36; 95% CI: 1.06 to 1.76). According to the multiple-adjusted model, pregnant women with anemia had a 38% higher risk of having children with low/insufficient weight at birth than the women without anemia (RR_**adjusted**_ = 1.38; 95% CI: 1.07 to 1.77; Table 4). The model was adjusted for the following confounders: maternal age, family income, urinary infection, parity, alcoholic beverage consumption during pregnancy and gestational body mass index. The quality of this model was considered good because the null hypothesis was rejected (p = 0.74).

**Table 4:** Crude and adjusted Relative Risk (RR) of the association between maternal anemia and low/insufficient birth weight with the corresponding 95% confidence intervals (95% CI).

Maternal anemia	Birth weight							
	<3000 g		≥3000 g		RR_crude_	95% CI p*	RR_adjusted_^†^	95% CI p*
	N	%	N	%				
Yes	57	36.8	98	63.2	1.36	1.06 to 1.76 0.02	1.38	1.07 to 1.77 0.01
No	126	27.0	341	73.0				

* p value: level of significance ≤ 0.05

^†^Adjusted by maternal age, family income, urinary infection, parity, alcoholic beverage consumption during pregnancy and gestational BMI. Model fit test (Hosmer-Lemeshow): p = 0.74.

The linear regression analysis showed that on average, there was a 21-g decrease (p = 0.03) in the weight of the newborn per 1 g/dl of reduced maternal hemoglobin during the gestational period. In the saturated model, which was adjusted for the above-mentioned confounders, there was a 0.20-g decrease (p = 0.05) in birth weight per 1 g/dl of maternal hemoglobin lost during pregnancy.

## Discussion

Despite a rigorous search of a large number of electronic databases, only two cohort studies were found that discussed the relationship between maternal anemia and insufficient birth weight [5, 6]. Studies addressing anemic exposure and low birth weight were more frequent. Regardless of the lack of previous investigations on the topic, the main results of the present investigation highlighted maternal anemia as a risk factor for low/insufficient birth weight, which was consistent with data from a previous study by Raisanen et al. (2014) [5]. Thus, these findings contribute to current knowledge concerning this important public health problem. Other findings from the present study using linear regression analysis confirmed the above association since women with hemoglobin depletion had children with significantly reduced birth weights. Only one investigation had contrasting findings [6], showing no association between maternal anemia and low/insufficient birth weight. In addition, the incidence of maternal anemia in the current sample was approximately 25%, corroborating the frequency found in other studies [17, 42–44]. Among these women, the incidence of children with low/insufficient birth weight was approximately 37%, whereas in children without anemia, the incidence was 27%.

The biological plausibility of the association between maternal anemia and low/insufficient birth weight is not fully understood [45, 46]. However, previous studies have argued that maternal anemia predisposes the fetus to intrauterine growth restriction and may consequently influence the birth weight [9, 47]. Physiologically, beginning during the middle of the second trimester of pregnancy, women produce an average of 30 to 40 ml of plasma per kilogram, corresponding to hypervolemia. However, when the number of hematological cells does not increase in parallel with this process, hemodilution occurs, and maternal anemia may develop [48].

Thus, low hemoglobin levels may stimulate changes in placental angiogenesis and favor fetal hypoxia. According to this theory, a reduction in nutrients and oxygen to the fetus due to deficits in placental transport may result from hemoglobin depletion. The potential framework of uterine growth restriction begins with a reduction in blood perfusion in the uterus, an elevation in vascular resistance and growth restriction of the trophoblastic surface, which is responsible for ejecting maternal arterial blood into the placenta. These events may result in the restriction of gas exchange within the maternal-fetal complex and, consequently, in low/insufficient birth weight [49].

Within the scope of the present investigation, the findings of only one study clearly point to the association between maternal anemia and a birth weight of less than 3000 g [5] since this exposure is often studied only in terms of its relationship with low birth weight [50–52]), without the inclusion of insufficient weight.

Multiple investigations have shown that maternal anemia is associated with low birth weight [53–55]. Conversely, several other studies that included only low birth weight as the outcome refuted the hypothesis under investigation [23, 56, 57], making the association controversial. Although discussions about the damage from insufficient birth weight have been carried out for more than 30 years, few studies have evaluated the relationship between insufficient weight and various undesirable gestational events [8–10, 58], emphasizing only the extreme ranges of birth weight, such as low weight and macrosomia [50–52].

A similarity does exist between low birth weight and insufficient weight, but the former is considered more serious than the latter for the newborn. However, the unfavorable effect produced by insufficient birth weight cannot be ignored since this condition may contribute to inadequate cognitive development and infant growth and increase the morbidity and mortality of this age group [3].

The findings of the present investigation are relevant in that they contribute to a better understanding of the importance of insufficient birth weight to pediatric health, and several method-related parameters, such as the sample size, diagnostic criteria for maternal anemia and treatment of confounders, should be carefully evaluated when comparing these results to those of previous studies on the topic.

The sample size of the present study exceeded the minimum calculated sample size to estimate the effect measurement. The total number of pregnant women involved in the study by Mesa et al. (2012) [6] was approximately half of that employed in the present investigation and may explain the non-association, which was probably due to the lack of power in the study, found between maternal anemia and low/insufficient birth weight. However, Raisanen et al. (2014) [5] evaluated a large sample comprising 290,622 pregnant women and found a positive association, corroborating the findings of the present study.

Regarding the diagnosis of anemia in the present investigation, to improve internal validity, a laboratory test was used to define maternal anemia, establishing a hemoglobin reference value <11 g/dl and confirming the result with a hematocrit <33%. These criteria are recommended by the World Health Organization [32]. Mesa et al. (2012) [6] used the same criteria, although the authors did not confirm the diagnosis of maternal anemia by hematocrit. However, Raisanen et al. (2014) [5] defined anemia using only the information contained in the hospital chart.

The subclassification of maternal anemia is highly valuable to ensure the adequate monitoring of pregnant women. For example, the diagnosis of iron deficiency anemia may facilitate therapy for the disease with iron supplementation. Increasingly, iron deficiency anemia has been classified as maternal anemia since multiple reports in the literature support the hypothesis that the primary cause of anemia in pregnant women is ferritin deficiency [17, 59–62]. This notion conflicts with the findings presented in this study because most women with the anemia were diagnosed with anemia of chronic disease (18.9%), with a low frequency of iron deficiency anemia (6.0%), thus corroborating other studies [63–65].

Regarding severity levels, the participants in this study had a higher frequency of mild anemia. This severity was expected because much of the research converges in this direction [60, 66–69].

In the present investigation, the effect measurement was adjusted by confounders, due to knowledge of the possible effects of these covariables on both the exposure factor and the outcome. This criterion was also used in the study by Raisanen et al. (2014) [5], which corroborated the findings of this study. In contrast, Mesa et al. (2012) [6] did not adjust for the confounding covariables, and this lack of adjustment may explain why their findings contrasted the association verified in the present research.

According to the conceptual framework adopted here and the multicausality involved in the association between maternal anemia and low/insufficient birth weight, the following covariables were considered in the adjustment of the final model: maternal age, family income, urinary infection, parity, alcoholic beverage consumption during pregnancy and gestational BMI.

Unfavorable socioeconomic-demographic factors can influence both the exposure and the gestational outcome of interest. Maternal age, in its extreme age ranges, is a classical confounding variable because younger women do not have complete biological maturity for gestation and because older women are more likely to have comorbidities, such as maternal anemia [25, 64]). Regarding family income, pregnant women who have lower purchasing power are more vulnerable in terms of poor living conditions and, consequently, health [43, 55]).

Infectious processes, such as urinary infection, influence the metabolism of new hemoglobin, leading to the development of maternal anemia [16], and intrauterine growth restriction, contributing to low/insufficient birth weight. Another relevant factor is parity since multiparity is a condition that can favor both low/insufficient birth weight and maternal anemia [25, 43].

Alcoholic beverage consumption during pregnancy can cause inflammatory disorders that restrict intrauterine growth, a contributing factor to low/insufficient birth weight [69, 70]. Alcohol consumption can also compromise caloric intake, making it insufficient and, consequently, favoring the development of maternal anemia [69, 70]. Women with inadequate nutrition are susceptible to both maternal anemia and having low/insufficient-birth-weight children, probably due to the poor dietary intake of essential micronutrients during pregnancy [43, 70].

Regarding the limitations of this investigation, the self-reported information may have produced calibration bias. Although the sample is representative of the urban population of the municipality investigated, caution should be exercised when interpreting the results by extrapolating them to other locations that do not have a population group that is similar to the one in this study. Other limitation is the exclusion of preterm births, since the source of information in Brazil about gestational age may be distorted, due to the absence of standardization of criteria used to define preterm birth. On the other hand, the low birth weight as outcome gave higher quality to the measurement due to its standardized form of collection [29, 71]. Regarding the definition of anemia, the criterion used was that standard, known worldwide and suggested by the WHO [32], which does not take into account the gestational trimester. As in any other investigation, the possibility of residual confounding remains since some factors may not have been measured in the present study.

Finally, this research can contribute information that has not previously been elucidated toward confirming the hypothesis. The temporality of the events, namely, the order of the exposure relative to the outcome, is established since the laboratory tests for the diagnosis of maternal anemia preceded delivery and birth. Possible sample losses were measured prior to the study, which assures the representativeness and power of the sample size and the reliability of the presented findings. The adoption of these criteria also minimizes the possibility of a spurious association. Furthermore, the use of validated instruments by the researchers, who were previously trained, strengthens the internal validity of this study.

Based on the method employed and the limitations described, the exposure investigated was confirmed as a risk factor for low/insufficient birth weight. Given the findings, a far-reaching measure in public health would be the implementation of healthcare actions for the prevention and control of maternal anemia, aiming to reduce unfavorable gestational outcomes.
